# Knowledge–practice concordance and discrepancies in very old adults

**DOI:** 10.3389/fmed.2026.1891660

**Published:** 2026-08-24

**Authors:** Aline Schönenberg, Jenny M. Ruttloff, Konstantin G. Heimrich, Tino Prell

**Affiliations:** Department of Geriatrics, Jena University Hospital, Jena, Germany

**Keywords:** health-related knowledge, internal control experience, knowledge–practice discrepancy, self-reported practice, very old adults

## Abstract

**Background:**

Health-related knowledge is often considered a prerequisite for health behavior, but knowledge may not always translate into everyday practice. This study examined concordance and directional discrepancies between self-reported health-related knowledge and practice among very old adults.

**Methods:**

We analyzed data from 2,919 participants aged 80 years and older from the nationally representative Old Age in Germany (D80+) study (CATI Module 2). Health-related knowledge and self-reported health-related practice were assessed using ordinal self-report items. A positive knowledge–practice discrepancy was defined as reported knowledge exceeding reported practice. CATI2-calibrated complex-survey descriptive analyses were used to examine knowledge–practice patterns. Complex-survey logistic regression models examined factors associated with the presence of a positive discrepancy, while linear models examined its magnitude as a secondary outcome. Exploratory age-, sex-, and education-adjusted models compared the health, functional, and psychosocial profiles of participants with and without a positive discrepancy.

**Results:**

Most participants reported the highest category of health-related knowledge (74.2, 95% CI 71.8 to 76.5%) and self-reported practice (73.7, 95% CI 71.3 to 76.0%). Knowledge and practice were concordant in 76.5% of participants (95% CI 74.4 to 78.6%), whereas 12.2% showed a positive knowledge–practice discrepancy (95% CI 10.5 to 13.9%). In fully adjusted complex-survey models, higher internal control experience was associated with lower odds of a positive knowledge–practice discrepancy and with a smaller discrepancy magnitude. In exploratory health-profile analyses, positive discrepancy status was associated with lower ADL and IADL scores, more depressive symptoms, lower life satisfaction, more negative aging experiences, and lower internal control experience; these associations remained significant after false-discovery-rate correction.

**Conclusion:**

In very old adults, knowledge and practice were generally concordant, while a minority reported more knowledge than practice. Positive discrepancy status co-occurred with a less favorable functional and psychosocial profile, and internal control experience was the most consistent cross-sectional correlate. Longitudinal and behavior-specific studies are needed to clarify the direction and practical relevance of these associations.

## Introduction

Health-related knowledge is commonly regarded as a prerequisite for informed health behavior, self-management, and adherence to preventive or therapeutic recommendations (1, 2). This assumption is particularly relevant in later life, when individuals are often confronted with multimorbidity, functional limitations, polypharmacy, and repeated interactions with health-care systems. However, knowledge alone does not necessarily translate into action (2, 3). Discrepancies between knowledge and behavior have been discussed under terms such as the “knowing–doing gap” or “knowledge–practice gap” in behavioral science and health-care research (4, 5). The concept is clinically relevant because the benefits of health information, counseling, and education depend on whether knowledge can be enacted in everyday life (6). Thus, an important question is not only whether individuals report sufficient knowledge, but also whether reported practice is consistent with that knowledge.

Very old adults aged 80 years and above represent a particularly relevant population in the context of knowledge–practice discrepancies, as adherence to health-related recommendations may have direct implications for disease control, functional ability, health-care utilization, and survival (7). Previous research has shown that non-adherence to medication and health recommendations is common in chronic disease populations and is associated with adverse outcomes (8–12). Many individuals in this age group have accumulated substantial health-related experience and may be well aware of general recommendations regarding medication use, physical activity, nutrition, prevention, or disease management. At the same time, consistency between perceived knowledge and self-reported daily practice may become more difficult when functional limitations, pain, multimorbidity, depressive symptoms, loneliness, or reduced perceived control interfere with action. Barriers to adherence and health behavior change in later life have been described across cognitive, functional, emotional, and social domains (8, 9, 13–16). Health behavior change also depends on motivational and reward-related mechanisms, as well as on bio-psychological and socio-environmental resources that support or hinder action (17, 18).

Importantly, a positive knowledge–practice discrepancy in very old age should not be equated with a general lack of information, objective non-adherence, or failure to implement a specific recommendation. The relevant empirical question is more specific: can a subgroup be identified in which knowledge exceeds self-reported practice, and which characteristics are associated with this response pattern? Such a response pattern may be compatible with barriers to acting on perceived knowledge, but it may also reflect response tendencies, differences in item interpretation, or other unmeasured factors. Identifying correlates of this pattern may generate hypotheses about the roles of capability, opportunity, motivation, and perceived agency in very old age (1, 16).

Several domains may influence this implementation process. Functional physical and cognitive capacity is likely to be relevant because even well-understood recommendations may be difficult to follow when basic or instrumental activities of daily living are impaired (15, 19). Clinical factors such as pain and multimorbidity may further complicate the enactment of health-related behaviors (15, 16). Psychosocial factors may be equally important. Depressive symptoms can reduce motivation and self-management capacity (20), while loneliness may limit daily structure and social support for health-related routines (21). In addition, views and experiences of aging may influence whether individuals perceive health-related action as worthwhile and feasible. Views on aging have been linked to health outcomes across adulthood and late life, suggesting that aging-related self-perceptions may shape health behavior and adaptation processes (22). Finally, internal control experience may be relevant to knowledge–practice alignment: individuals who perceive greater personal influence over their health and daily life may report greater consistency between knowledge and practice (23).

Using data from the nationally representative “Old Age in Germany” study (D80+), the present study examines self-reported health-related knowledge, self-reported health-related practice, and directional discrepancies between the two among adults aged 80 years and older (24). We defined a positive knowledge–practice discrepancy as reported knowledge exceeding self-reported practice.

The study addressed the following research questions:

*RQ1:* How are health-related knowledge, practice, and their concordance and discordance patterns distributed among adults aged 80 years and older?

*RQ2:* What proportion of very old adults shows a positive knowledge–practice discrepancy, defined as reported knowledge exceeding practice?

*RQ3:* Which sociodemographic, clinical-functional, and psychosocial factors are associated with the presence and magnitude of a positive knowledge–practice discrepancy?

*RQ4:* Do adults with a positive knowledge–practice discrepancy show a less favorable health, functional, or psychosocial profile than those without a positive discrepancy?

## Methods

### Sample

We used data from the ‘Old Age in Germany (D80+)’ study (CATI2 module), which is a large, nationally representative sample of individuals aged 80 and over living in Germany (24) (Hohes Alter in Deutschland (D80+): Deutsches Zentrum für Altersfragen). The study encompassed participants living in both the community and institutional settings, such as nursing homes. The D80 + study was performed by the University of Cologne in partnership with the Cologne Center for Ethics, Rights, Economics, and Social Sciences of Health (CERES) and the German Center of Gerontology (DZA). Approval for the D80 + study was granted by the ethical board of the Medical Faculty at the University of Cologne (Protocol #: 19-1387_1).

Of the 10,578 participants in the original D80 + Scientific Use File, 3,233 were included in CATI Module 2. Valid responses to the health-related knowledge item were available for 3,069 participants. Among these, 118 participants selected the lowest knowledge category (“never”) and were therefore not administered the practice item according to the questionnaire skip logic. Practice data were missing for a further 32 eligible participants. Consequently, 2,919 participants had valid information on both knowledge and practice and formed the analytical sample for the descriptive knowledge–practice analyses. All 2,919 participants had valid CATI2 calibrated weights, primary sampling unit identifiers, and stratum information. The analytical sample included 2,627 target-person interviews (90.0%) and 292 proxy interviews (10.0%). The derivation of the analytical sample and the model-specific sample sizes are summarized in Supplementary Table S1.

### Variables of interest

Health-related knowledge (*knowledge*) was assessed by asking how often respondents know what to do to maintain or restore their health (“How often do you know what you need to do to stay healthy, get healthy again or strengthen your health?”). Responses were coded from 1 (never), 2 (rather rarely), 3 (sometimes) to 4 (frequently), with higher values indicating greater perceived health-related knowledge.

Self-reported health-related practice (practice) was assessed as a follow-up to the knowledge item. Participants who reported at least some health-related knowledge were subsequently asked how often they acted accordingly; the brief questionnaire wording was “How often do you comply?.” In the context of the preceding knowledge question, this item was interpreted as referring broadly to acting on perceived knowledge about maintaining, restoring, or strengthening health. The item did not refer to a specific behavior, recommendation, or clinical domain. It was administered only to participants in knowledge categories 2 to 4. Responses were coded from 1 (never), 2 (rather rarely), 3 (sometimes), to 4 (frequently), with higher values indicating more frequent self-reported practice.

CATI Module 2 included both target-person and proxy interview versions of the knowledge and practice items. Proxy responses were retained in the main analysis because they formed part of the D80 + Scientific Use File and used corresponding proxy wording. A sensitivity analysis excluding proxy interviews was conducted.

The primary outcome was the *presence* of a positive knowledge–practice discrepancy, defined as reported knowledge exceeding self-reported practice. As a secondary outcome, positive-discrepancy magnitude was calculated as max(knowledge − practice, 0), with values of zero assigned to participants without a positive discrepancy. This operationalization was used as a pragmatic comparison of two similarly coded ordinal items and was not interpreted as a validated measure of objective implementation or adherence.

Throughout the manuscript, “knowledge” and “practice” refer to the two self-report items described above. “Practice” does not denote objectively observed behavior.

### Covariates

Covariates were selected *a priori* based on clinical relevance, previous literature, and availability in the dataset and grouped into sociodemographic, clinical-functional, and psychosocial domains. Sociodemographic covariates included age, sex, and education. Clinical-functional covariates included pain, multimorbidity, basic activities of daily living (ADL), and instrumental activities of daily living (IADL). Psychosocial covariates included loneliness, depressive symptoms, life satisfaction, positive and negative aging experiences, and internal and external control experience. Higher ADL and IADL scores indicate greater functional independence; higher scores on loneliness, depressive symptoms, positive and negative aging experiences, and control experience indicate higher levels of the respective construct. Details about the variables are given in Supplementary Table S2.

### Statistical analyses

All analyses were conducted in R version 4.5.1. The analytical sample was restricted to participants with valid information on both health-related knowledge and self-reported health-related practice. Because the knowledge and practice items were administered in CATI Module 2, weighted descriptive and regression analyses used the CATI2 calibrated weighting factor (catii2kal) provided in the D80 + Scientific Use File (D80+ Dokumentation: Deutsches Zentrum für Altersfragen). Complex-survey analyses additionally incorporated community-level primary sampling units and survey strata. Unweighted values were used for the sample characteristics table to describe the analytical sample. Because randomized PSU codes could recur across strata, PSUs were specified as nested within strata. Variances and 95% confidence intervals were estimated using Taylor-series linearization in the R survey package.

To define the positive knowledge–practice discrepancy, we first calculated a directed difference score as knowledge minus practice. Based on this score, we derived one primary binary outcome and one secondary magnitude outcome. The primary binary outcome indicated the presence of a positive discrepancy and was coded 1 if knowledge exceeded practice and 0 otherwise. The secondary magnitude outcome was calculated as max(knowledge − practice, 0), with values of zero assigned to participants without a positive discrepancy.

For the ordinal sensitivity analysis, this score was categorized as 0, 1, or 2 or more response-category differences. Because the positive knowledge–practice discrepancy magnitude score was derived from two ordinal items and was bounded with a large proportion of zero values, the linear model was considered a secondary, pragmatic analysis. As a sensitivity analysis that did not assume equal spacing between discrepancy categories, we fitted a survey-weighted proportional-odds model. The outcome distinguished no positive discrepancy, a discrepancy of one response category, and a discrepancy of at least two response categories. Gap values of two and three were combined because a three-category discrepancy occurred in only two participants. The ordinal model used the same hierarchical covariate blocks and complex survey-design specification as the primary models. Because a Brant test that directly incorporates the complex survey design is not implemented for the survey-weighted proportional-odds model used here, we assessed the proportional-odds assumption diagnostically using a Brant test in the corresponding unweighted complete-case ordinal logistic model with the same outcome and covariate specification.

Descriptive analyses summarized the distribution of health-related knowledge, self-reported practice, and knowledge–practice patterns. Weighted proportions were calculated for knowledge categories, practice categories, exact concordance, positive discrepancies, and reverse discordance in which practice exceeded knowledge. A weighted cross-tabulation of knowledge and practice was additionally used to visualize concordance and discordance patterns.

To address RQ3, we examined factors associated with a positive knowledge–practice discrepancy using two complementary survey-weighted regression approaches. First, survey-weighted logistic regression models estimated associations with the presence of positive knowledge–practice discrepancy. Second, survey-weighted linear regression models estimated associations with the magnitude of positive knowledge–practice discrepancy. All regression models incorporated the calibrated analysis weight, primary sampling units, and survey strata. Because the randomized PSU identifiers could recur across strata, PSUs were specified as nested within strata. Standard errors, confidence intervals, and hypothesis tests were calculated using Taylor-series linearization and design-based degrees of freedom. Models were estimated using complete cases for the variables included in the respective model. Independent variables were entered hierarchically. Model 1 included age, sex, and education. Model 2 additionally included clinical and functional variables: pain, multimorbidity, ADL, and IADL. Model 3 additionally included psychosocial variables: loneliness, depressive symptoms, life satisfaction, positive aging experience, negative aging experience, internal control experience, and external control experience. Multicollinearity diagnostics did not indicate problematic collinearity in the fully adjusted model; all adjusted GVIF values were below 2. Global model significance was assessed using design-adjusted Wald tests.

To assess potential complete-case selection, participants included in the fully adjusted model were compared with those excluded because of missing covariate data using complex-survey-weighted descriptive statistics and standardized mean differences. Comparisons were based on observed values only, without treating missingness as a separate category (Supplementary Table S3). In an observed-only CATI2-calibrated comparison of participants included in and excluded from the fully adjusted model, group differences were small for all variables, with SMDs ranging from <0.01 to 0.14.

As a second sensitivity analysis, we examined the three directional knowledge–practice patterns separately. Two design-based logistic contrast models were fitted with exact concordance as the reference category: knowledge exceeding practice versus concordance, and practice exceeding knowledge versus concordance. Both models used the fully adjusted covariate set and accounted for calibrated weights, PSU clustering, and stratification.

To address RQ4, we conducted an exploratory health-profile analysis comparing participants with and without a positive knowledge–practice discrepancy. In these models, positive-discrepancy status was used as the independent variable, and selected health-related indicators were treated as outcomes. These outcomes included multimorbidity, ADL, IADL, depressive symptoms, life satisfaction, positive and negative aging experiences, and internal and external control experience. Survey-weighted linear regression models were estimated for each continuous outcome, adjusted for age, sex, and education. Regression coefficients indicate the adjusted mean difference between participants with and without positive knowledge–practice discrepancy. Negative coefficients indicate lower values in the positive knowledge–practice discrepancy group, whereas positive coefficients indicate higher values.

Regression models were estimated using complete cases for the variables included in each model. Missing covariate data were handled using complete-case analysis within each regression model. Within the knowledge–practice analytical sample, missingness was highest for depressive symptoms (9.8%), whereas missingness for all other covariates was below 3%. Consequently, complete-case sample sizes were 2,847 participants (97.5%) for Model 1, 2,708 (92.8%) for Model 2, and 2,324 (79.6%) for the fully adjusted Model 3. Variable-specific missingness and model-specific complete-case sample sizes are reported in Supplementary Table S4.

## Results

### Sample characteristics

The final knowledge–practice analytical sample therefore comprised 2,919 participants with a mean age of 85.65 years. The analytical sample included 292 proxy interviews (10.0%). The fully adjusted complete-case model included 225 proxy interviews (9.7%). Participants were moderately functionally independent. Most participants reported low to moderate loneliness, modest depressive symptom burden, and relatively high internal control experience. Detailed sample characteristics are shown in Table 1.

**Table 1:** Sample characteristics of the analytical sample.

Variable	Level	Overall
N		2,919
Age, mean (SD)		85.65 (4.10)
Sex, n (%)	Male	1,485 (50.9)
	Female	1,434 (49.1)
Education, n (%)	Lower secondary or below	384 (13.2)
	Upper secondary	1,421 (48.7)
	Tertiary	1,042 (35.7)
	Missing	72 (2.5)
Pain, n (%)	No pain	683 (23.4)
	Mild pain	808 (27.7)
	Moderate pain	911 (31.2)
	Severe pain	383 (13.1)
	Very severe pain	66 (2.3)
	Missing	68 (2.3)
Multimorbidity, mean (SD)		0.22 (0.14)
Basic ADL, mean (SD)		1.79 (0.38)
Instrumental ADL, mean (SD)		1.60 (0.52)
Loneliness, n (%)	Never or almost never	1,646 (56.4)
	Sometimes	1,020 (34.9)
	Mostly	151 (5.2)
	Always or almost always	32 (1.1)
	Missing	70 (2.4)
Depressive symptoms, mean (SD)		1.11 (1.19)
Life satisfaction, mean (SD)		7.56 (1.90)
Positive aging experience, mean (SD)		3.33 (0.71)
Negative aging experience, mean (SD)		2.71 (0.81)
Internal control experience, mean (SD)		3.27 (0.65)
External control experience, mean (SD)		1.92 (0.69)
Health-related knowledge, n (%)	Rather rarely	289 (9.9)
	Sometimes	413 (14.1)
	Frequently	2,217 (76.0)
Self-reported health-related practice, n (%)	Never	16 (0.5)
	Rather rarely	197 (6.7)
	Sometimes	486 (16.6)
	Frequently	2,220 (76.1)

Values are unweighted. ADL, activities of daily living; IADL, instrumental activities of daily living. Higher ADL and IADL scores indicate greater independence. Health-related knowledge and self-reported practice were each assessed on four-point ordinal scales. The category “Never” is absent for health-related knowledge in the analytical sample because practice was only assessed among participants reporting at least rare health-related knowledge. Positive knowledge–practice discrepancy was defined as knowledge exceeding self-reported practice.

### Health-related knowledge, self-reported practice, and positive knowledge–practice discrepancy

In complex-survey-weighted analyses, 74.2% of participants reported frequent health-related knowledge (95% CI 71.8 to 76.5%), and 73.7% reported frequent self-reported health-related practice (95% CI 71.3 to 76.0%). Exact concordance between the two response categories was observed in 76.5% of participants (95% CI 74.4 to 78.6%; Table 2).

**Table 2:** Complex-survey-weighted distribution of health-related knowledge, practice, and knowledge–practice patterns.

Variable	Category	Unweighted n	Weighted %	95% CI
Health-related knowledge	Rather rarely	289	11.2	9.3–13.1
	Sometimes	413	14.6	12.8–16.4
	Frequently	2,217	74.2	71.8–76.5
Practice	Never	16	0.7	0.2–1.2
	Rather rarely	197	8.3	6.4–10.3
	Sometimes	486	17.3	15.5–19.0
	Frequently	2,220	73.7	71.3–76.0
Knowledge–practice pattern	Knowledge = practice	2,225	76.5	74.4–78.6
	Knowledge > practice	345	12.2	10.5–13.9
	Practice > knowledge	349	11.3	9.8–12.8

Unweighted n indicates the observed number of participants in each category. Weighted percentages and 95% confidence intervals used the CATI2 calibrated weighting factor because the knowledge and practice items were administered in CATI Module 2. Estimates additionally accounted for primary sampling units and survey strata. Variances were estimated using Taylor-series linearization. Weighted counts are not shown because calibrated survey weights produce population-adjusted estimates that do not necessarily sum to the unweighted analytical sample size. The category “Never” is absent for health-related knowledge in the analytical sample because the practice item was administered only to participants reporting at least rare health-related knowledge. A positive knowledge–practice discrepancy was defined as knowledge exceeding self-reported practice.

**Figure 1:**
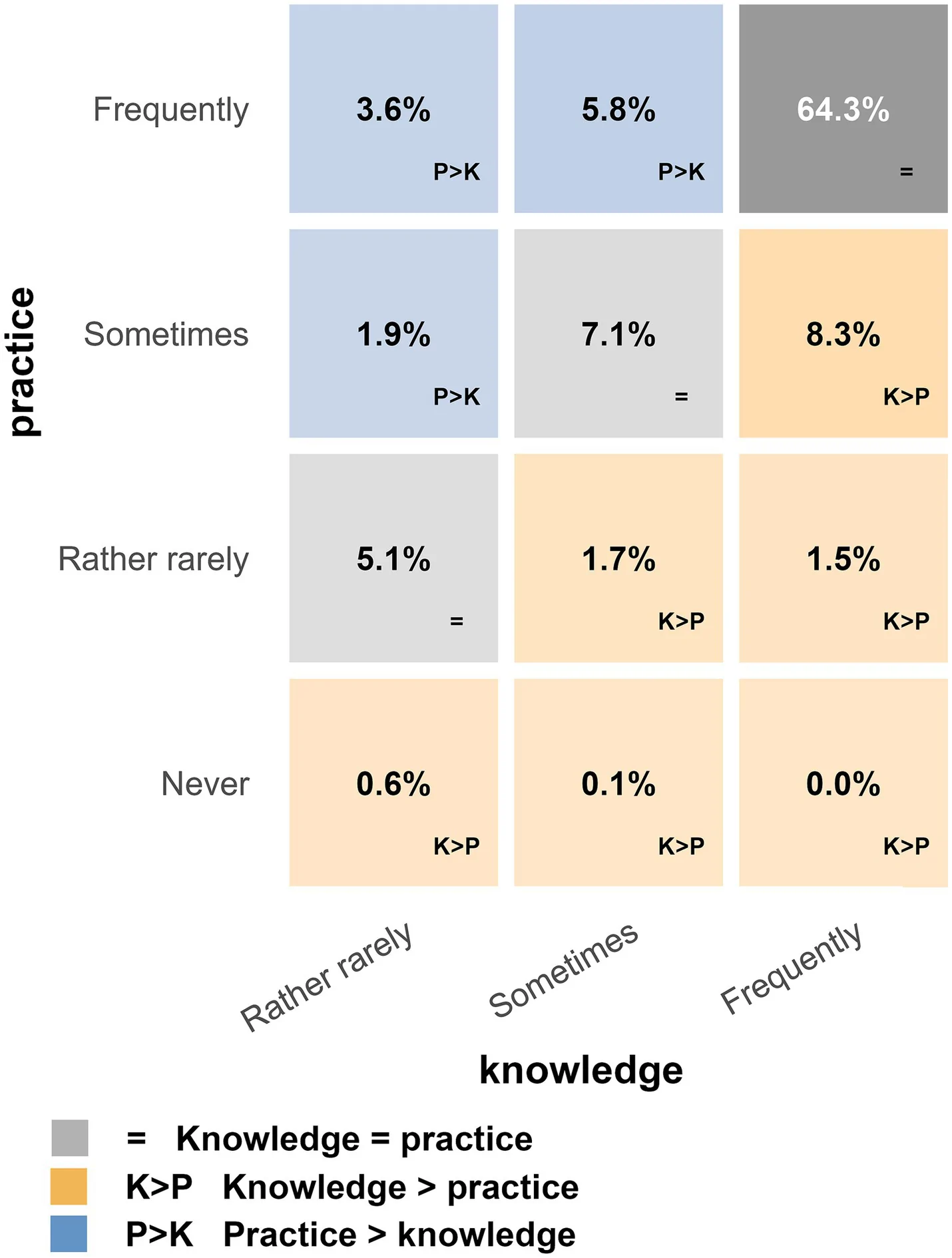
Complex-survey-weighted distribution of health-related knowledge and practice. Cells show the weighted percentage of the analytical sample in each combination of health-related knowledge and practice. Estimates used the CATI2 calibrated weighting factor and accounted for primary sampling units and survey strata. Gray cells indicate exact concordance between knowledge and practice, orange cells indicate a positive knowledge–practice discrepancy (knowledge > practice), and blue cells indicate reverse discordance (practice > knowledge). The symbols “=,” “K ≥ P,” and “P ≥ K” provide an additional non-color-based indication of the respective pattern. Color intensity reflects the weighted frequency of each cell. The knowledge category “Never” is absent because the practice item was administered only to participants reporting at least rare health-related knowledge.

A positive knowledge–practice discrepancy, defined as knowledge exceeding self-reported practice, was present in 12.2% of participants (95% CI 10.5 to 13.9%). The reverse pattern, in which practice exceeded knowledge, was observed in 11.3% (95% CI 9.8 to 12.8%; Table 2). The largest cell in the weighted cross-tabulation comprised participants reporting both frequent knowledge and frequent practice (Figure 1).

### Factors associated with positive knowledge–practice discrepancy

Complete-case sample sizes were 2,847 participants for Model 1, 2,708 for Model 2, and 2,324 for the fully adjusted Model 3. The corresponding numbers of participants with a positive knowledge–practice discrepancy were 335, 321, and 280.

In the fully adjusted logistic model, internal control experience was the only statistically significant correlate of discrepancy presence. Higher internal control experience was associated with lower odds of reporting knowledge that exceeded self-reported practice (OR 0.69, 95% CI 0.53 to 0.89; *p* = 0.005). None of the other sociodemographic, clinical-functional, or psychosocial variables was independently associated with discrepancy presence (Table 3; F[20,1,426] = 1.87; *p* = 0.011).

**Table 3:** Fully adjusted complex-survey models for positive knowledge–practice discrepancy.

Predictor	Discrepancy presence OR (95% CI), *p*	Discrepancy magnitude B (95% CI), *p*
Age	1.01 (0.97–1.06), 0.547	0.002 (−0.004 to 0.008), 0.481
Female	0.92 (0.63–1.34), 0.670	−0.015 (−0.060 to 0.030), 0.512
Education: upper secondary	0.93 (0.58–1.48), 0.747	−0.007 (−0.068 to 0.053), 0.815
Education: tertiary	0.99 (0.59–1.65), 0.955	0.011 (−0.058 to 0.079), 0.763
Pain: mild	0.86 (0.51–1.45), 0.567	−0.016 (−0.077 to 0.044), 0.599
Pain: moderate	0.91 (0.54–1.53), 0.709	−0.012 (−0.072 to 0.048), 0.691
Pain: severe	1.21 (0.64–2.28), 0.552	0.031 (−0.049 to 0.110), 0.453
Pain: very severe	0.83 (0.27–2.54), 0.747	−0.040 (−0.169 to 0.088), 0.540
Multimorbidity	0.55 (0.14–2.14), 0.390	−0.083 (−0.242 to 0.076), 0.307
Basic ADL	0.87 (0.47–1.62), 0.662	−0.029 (−0.122 to 0.065), 0.546
Instrumental ADL	1.19 (0.69–2.04), 0.532	0.016 (−0.056 to 0.088), 0.664
Loneliness: sometimes	1.09 (0.77–1.54), 0.645	0.017 (−0.026 to 0.060), 0.434
Loneliness: mostly	1.08 (0.54–2.16), 0.820	0.015 (−0.088 to 0.118), 0.772
Loneliness: always or almost always	0.61 (0.16–2.33), 0.470	−0.086 (−0.235 to 0.063), 0.259
Depressive symptoms	1.12 (0.94–1.34), 0.192	0.018 (−0.007 to 0.043), 0.151
Life satisfaction	0.94 (0.85–1.05), 0.269	−0.011 (−0.028 to 0.007), 0.225
Positive aging experience	1.00 (0.77–1.29), 0.988	0.005 (−0.026 to 0.036), 0.734
Negative aging experience	1.23 (0.96–1.58), 0.096	0.015 (−0.015 to 0.044), 0.326
Internal control experience	0.69 (0.53–0.89), 0.005	−0.055 (−0.098 to −0.012), 0.012
External control experience	0.99 (0.76–1.30), 0.964	−0.004 (−0.041 to 0.033), 0.820

Estimates are from fully adjusted complex-survey regression models using the CATI2 calibrated weighting factor because the knowledge and practice items were administered in CATI Module 2. Models additionally accounted for primary sampling units and survey strata; variances were estimated using Taylor-series linearization. The logistic model predicted the presence of a positive knowledge–practice discrepancy, defined as reported health-related knowledge exceeding self-reported health-related practice. The linear model predicted positive-discrepancy magnitude, calculated as max (knowledge − practice, 0), and should be interpreted as a secondary, pragmatic analysis because the outcome was derived from two ordinal items, was bounded, and contained a high proportion of zero values. Both models included age, sex, education, pain, multimorbidity, basic ADL, instrumental ADL, loneliness, depressive symptoms, life satisfaction, positive and negative aging experiences, and internal and external control experience. The fully adjusted models included 2,324 participants; 280 participants had a positive knowledge–practice discrepancy. The fully adjusted logistic model was significant overall (F[20,1,426] = 1.87; *p* = 0.011), whereas the corresponding linear model did not reach conventional statistical significance overall (F[20,1,426] = 1.44; *p* = 0.096). Reference categories were male sex, lower secondary education or below, no pain, and loneliness “never or almost never.” Odds ratios below 1 indicate lower odds of a positive discrepancy; negative linear coefficients indicate a smaller positive-discrepancy magnitude. ADL, activities of daily living; IADL, instrumental activities of daily living.

The corresponding linear model showed a consistent association between internal control and discrepancy magnitude (B − 0.055, 95% CI − 0.098 to −0.012; *p* = 0.012), although the overall model test did not reach conventional statistical significance (F[20,1,426] = 1.44; *p* = 0.096).

Across the hierarchical models, sociodemographic and clinical-functional variables were not jointly associated with discrepancy presence or magnitude. The fully adjusted logistic model reached statistical significance only after psychosocial variables were included, with internal control experience emerging as the only significant individual correlate in the fully adjusted logistic and linear models. Full hierarchical results are presented in Supplementary Tables S5, S6, respectively.

The ordinal sensitivity analysis yielded a consistent result. In the fully adjusted proportional-odds model, higher internal control experience was associated with lower odds of belonging to a higher positive discrepancy category (OR 0.68, 95% CI 0.52 to 0.89; *p* = 0.005). None of the other sociodemographic, clinical-functional, or psychosocial variables was statistically significant (Supplementary Table S7). The diagnostic Brant test in the corresponding unweighted complete-case ordinal logistic model did not indicate evidence of a global violation of the proportional-odds assumption (χ^2^ = 12.10, df = 20, *p* = 0.913). The ordinal model was therefore retained as a supportive sensitivity analysis.

The directional pattern analysis further supported the main result. When participants with knowledge exceeding practice were compared only with those reporting exact concordance, higher internal control experience remained associated with lower odds of a positive knowledge–practice discrepancy (OR 0.66, 95% CI 0.51 to 0.85; *p* = 0.001). In this contrast, higher negative aging experience was also associated with higher odds of knowledge exceeding practice (OR 1.31, 95% CI 1.02 to 1.68; *p* = 0.038; Supplementary Table S8).

The reverse-discordance contrast showed a different pattern. Compared with exact concordance, practice exceeding knowledge was less common among women and participants with higher multimorbidity, and was more common among participants with more negative aging experiences. Higher internal control experience was also associated with lower odds of reverse discordance (OR 0.71, 95% CI 0.54 to 0.94; *p* = 0.017; Supplementary Table S8). These results suggest that internal control may be associated with closer knowledge–practice alignment rather than exclusively with the absence of positive knowledge–practice discrepancy.

In a sensitivity analysis excluding proxy interviews, the internal-control estimate remained in the same direction but was attenuated and no longer statistically significant for discrepancy presence (OR 0.82, 95% CI 0.61 to 1.09; *p* = 0.174) or discrepancy magnitude (B − 0.024, 95% CI − 0.060 to 0.012; *p* = 0.200; Supplementary Table S9).

### Health profile of participants with positive knowledge–practice discrepancy

We next explored whether positive-discrepancy status was associated with a less favorable health, functional, and psychosocial profile. In exploratory age-, sex-, and education-adjusted complex-survey models, positive-discrepancy status was associated with lower basic ADL scores (B − 0.145, 95% CI − 0.250 to −0.040; q = 0.012) and lower IADL scores (B − 0.154, 95% CI − 0.274 to −0.033; q = 0.019). It was also associated with more depressive symptoms (B 0.392, 95% CI 0.186 to 0.599; q < 0.001), lower life satisfaction (B − 0.638, 95% CI − 0.998 to −0.277; q = 0.002), more negative aging experiences (B 0.323, 95% CI 0.162 to 0.485; q < 0.001), and lower internal control experience (B − 0.295, 95% CI − 0.475 to −0.115; q = 0.003). No FDR-adjusted associations were observed for multimorbidity, positive aging experience, or external control experience (unadjusted *p*-values and FDR-adjusted q-values are reported in Table 4).

**Table 4:** Exploratory health, functional, and psychosocial profile of participants with a positive knowledge–practice discrepancy.

Outcome	N	B (95% CI)	*p*	q
Multimorbidity	2,793	0.009 (−0.013 to 0.030)	0.429	0.429
Basic ADL	2,846	−0.145 (−0.250 to −0.040)	0.007	0.012
Instrumental ADL	2,818	−0.154 (−0.274 to −0.033)	0.013	0.019
Depressive symptoms	2,570	0.392 (0.186 to 0.599)	<0.001	<0.001
Life satisfaction	2,778	−0.638 (−0.998 to −0.277)	<0.001	0.002
Positive aging experience	2,822	−0.117 (−0.269 to 0.035)	0.131	0.147
Negative aging experience	2,815	0.323 (0.162 to 0.485)	<0.001	<0.001
Internal control experience	2,833	−0.295 (−0.475 to −0.115)	0.001	0.003
External control experience	2,830	0.141 (−0.008 to 0.290)	0.063	0.081

Estimates are from separate survey-weighted linear regression models adjusted for age, sex, and education. Each model accounted for calibrated weights, primary sampling units, and survey strata. The independent variable was the presence of a positive knowledge–practice discrepancy, defined as knowledge exceeding self-reported practice. Coefficients represent adjusted mean differences compared with participants without a positive discrepancy. Negative coefficients indicate lower values in the positive-discrepancy group and positive coefficients indicate higher values. q-values were calculated using the Benjamini–Hochberg false discovery rate procedure across the nine exploratory outcomes. ADL, activities of daily living; IADL, instrumental activities of daily living.

## Discussion

### Principal findings

In this analytical sample drawn from the nationally representative D80 + study, health-related knowledge and self-reported health-related practice were largely concordant. Most participants reported high levels of both knowledge and practice, and a positive knowledge–practice discrepancy was present in only a minority of participants. Thus, the findings do not indicate widespread self-reported discordance between knowledge and practice in this analytical sample. Rather, they identify knowledge exceeding practice as one specific discordance pattern affecting a smaller, but potentially relevant subgroup.

Across the primary CATI2-calibrated complex-survey models, internal control experience emerged as the most consistent correlate of the positive knowledge–practice discrepancy. Higher internal control experience was associated with both lower odds of having a positive discrepancy and a smaller discrepancy magnitude. However, the proxy-excluded sensitivity analysis attenuated this association, indicating that the finding should be interpreted cautiously. In contrast, sociodemographic factors, pain, multimorbidity, functional capacity, loneliness, depressive symptoms, life satisfaction, and aging experiences were not independently associated with the positive discrepancy after mutual adjustment in the main fully adjusted models.

### Interpretation of the knowledge–practice pattern

A first important finding is that most very old adults reported frequent health-related practice. This is relevant because older adults are often implicitly framed as a population at high risk of non-adherence or insufficient implementation of health recommendations. Our results suggest a more nuanced interpretation. At least in self-report, many adults aged 80 years and older appear to perceive themselves as acting in accordance with their health-related knowledge.

The observed concordance may reflect accumulated health experience, repeated exposure to medical advice, or adaptation to chronic disease management over the life course. It may also reflect selective participation, because individuals who respond to survey items on health knowledge and practice may be more cognitively, functionally, or motivationally able to engage with health-related recommendations. Therefore, the high level of reported practice should not be interpreted as objective evidence of universal adherence, but rather as an important self-reported pattern within the analytical sample (25).

At the same time, approximately one in nine participants showed the specific pattern of higher knowledge than practice. This subgroup is conceptually relevant because respondents reported greater knowledge than practice, although the broad items do not establish which specific behaviors were known or implemented. This pattern raises the hypothesis-generating question of what personal or contextual factors may contribute to closer alignment between perceived knowledge and self-reported practice (26, 27).

### Internal control experience and knowledge–practice alignment

The most consistent cross-sectional finding was the association of higher internal control experience with both lower odds and a smaller magnitude of a positive knowledge–practice discrepancy. The effect estimates were modest but remained statistically significant after accounting for sociodemographic, clinical-functional, and psychosocial characteristics. The association was also reproduced in an ordinal sensitivity analysis that treated discrepancy magnitude as an ordered outcome rather than as an equally spaced numerical score. The proxy-excluded sensitivity analysis provides an important qualification. Although the internal-control estimate remained in the same direction after excluding proxy interviews, the association was attenuated and no longer statistically significant. This suggests that the main finding is not fully independent of respondent type and should be interpreted as a cross-sectional association observed in the full analytical sample, which included both target-person and proxy interviews.

This pattern is compatible with the hypothesis that perceived personal influence may be relevant to knowledge–practice alignment. However, the cross-sectional data cannot establish whether stronger internal control facilitates health-related practice, results from successful everyday routines, or reflects other unmeasured personal and environmental resources. Older adults with stronger internal control beliefs may be more likely to perceive recommendations as actionable, to initiate routines, and to persist despite barriers (28–30).

This interpretation is compatible with broader theories of health behavior change, which emphasize that information alone is rarely sufficient. Behavioral implementation also requires motivation, perceived capability, reinforcement, and environmental support (26, 31, 32). In this framework, internal control experience may be interpreted as an agency-related correlate of knowledge–practice alignment. One possible interpretation is that individuals with greater perceived control may also perceive health-related recommendations as more actionable and their routines as more consistent with what they know. This interpretation remains speculative.

The directional sensitivity analysis adds an important nuance. Higher internal control experience was associated not only with lower odds of knowledge exceeding practice, but also with lower odds of the reverse pattern in which practice exceeded knowledge. Internal control may therefore be related more broadly to consistency between perceived knowledge and self-reported practice rather than specifically to the translation of knowledge into action. This interpretation should remain tentative because both measures were broad single self-report items and the cross-sectional design does not establish directionality.

Internal control warrants further investigation as an agency-related correlate in longitudinal and behavior-specific studies. Such studies could examine whether perceived control, self-efficacy, goal setting, and problem-solving contribute to subsequent knowledge–practice alignment (27, 33, 34). Future intervention studies could then test whether practical, context-sensitive support improves behavior-specific implementation in very old adults.

### Functional and psychosocial burden: predictors versus profile markers

Several clinical, functional, and psychosocial factors that were expected to be associated with positive knowledge–practice discrepancy were not independent correlates in the fully adjusted predictor models. These included ADL and IADL function, pain, multimorbidity, loneliness, depressive symptoms, life satisfaction, and aging experiences. This does not imply that these domains are irrelevant for positive knowledge–practice discrepancy. Rather, it suggests that their independent associations were attenuated after mutual adjustment, likely because functional limitations, depressive symptoms, life satisfaction, aging experiences, and perceived control are closely interrelated dimensions of vulnerability in very old age (30).

This interpretation is supported by the health-profile analysis. When positive knowledge–practice discrepancy was examined as a group marker, participants with a positive knowledge–practice discrepancy had a less favorable functional and psychosocial profile. They showed lower ADL and IADL scores, more depressive symptoms, lower life satisfaction, more negative aging experiences, and lower internal control experience. These associations remained statistically significant after FDR correction. However, these findings should not be interpreted as evidence that the discrepancy caused poorer functioning or psychosocial burden. The analyses were cross-sectional, each outcome was modeled separately, and several of the measures are conceptually interrelated. The results therefore identify a pattern of co-occurring vulnerability rather than directional effects.

These findings therefore highlight an important distinction. The predictor models addressed which factors were independently associated with the presence and magnitude of positive knowledge–practice discrepancy, whereas the health-profile analysis examined whether the positive knowledge–practice discrepancy identifies a subgroup with increased vulnerability. Taken together, the exploratory results indicate that positive-discrepancy status co-occurred more clearly with functional and psychosocial vulnerability than with multimorbidity. This pattern is compatible with internal control reflecting a broader dimension of perceived agency, but the cross-sectional design does not establish a mediating or explanatory mechanism.

### Implications for future research

The findings support several cautious implications. First, broad health-related knowledge and self-reported practice should not be treated as interchangeable constructs. In clinical assessment, it may therefore be useful to ask separately what an older person understands and what they report being able to do in everyday life. Second, the association with internal control suggests that perceived agency deserves further investigation, but the present data do not establish it as an intervention target. Third, because the items did not refer to specific behaviors, the results cannot directly support recommendations for medication adherence, physical activity, nutrition, or disease management. Future studies should use behavior-specific measures and longitudinal designs to determine whether discrepancies between knowledge and practice identify individuals who benefit from additional support (31, 33, 35).

### Strengths

This study has several strengths. It used data from a large population-based study of adults aged 80 years and older, a group that is often underrepresented in health behavior research. The analysis focused specifically on the direction of the knowledge–practice discrepancy rather than treating all discordance as equivalent. This distinction allowed us to separate knowledge exceeding practice from the reverse pattern of practice exceeding knowledge. Another strength is the broad set of sociodemographic, clinical-functional, and psychosocial predictors. By including sociodemographic, clinical-functional, and psychosocial variables, the study could examine whether positive knowledge–practice discrepancy was more closely related to health limitations, or psychological resources.

### Limitations

Several limitations should be considered. First, both core constructs were assessed using single, broad, ordinal self-report items. The knowledge item captured perceived general health-related knowledge, while the practice item assessed broad self-reported practice without specifying a particular recommendation, behavior, disease, or clinical context. The measures therefore do not establish objective knowledge, observed behavior, medication adherence, physical activity, nutrition, or actual fulfillment of medical recommendations. In addition, both items showed a high concentration in the highest response category, suggesting possible ceiling effects, shared response tendencies, or social desirability. The positive discrepancy was derived by subtracting two ordinal items, which assumes comparable category spacing and meaning across the knowledge and practice items. This pragmatic operationalization should therefore be interpreted cautiously, although the ordinal sensitivity analysis yielded consistent results. The practice item was administered only to participants who reported at least some health-related knowledge; participants selecting “never” for knowledge were excluded because practice was structurally missing. The findings therefore do not describe the full joint distribution of knowledge and practice in the original D80 + cohort. The cross-sectional design further precludes causal inference: internal control may contribute to knowledge–practice alignment, but it may also result from successful routines or reflect unmeasured personal and environmental resources. The health-profile analyses should likewise be interpreted as identifying co-occurring vulnerability rather than consequences of the discrepancy. Finally, the fully adjusted model used complete cases and included 79.6% of the analytical sample. Included and excluded participants were broadly similar on most observed characteristics, but residual selection bias cannot be ruled out. Because valid self-reported knowledge and practice data were required, individuals with severe cognitive impairment, communication difficulties, high care dependency, or reliance on proxy reporting may be underrepresented. Approximately 10% of the analytical sample consisted of proxy interviews. Although the D80 + instrument used corresponding proxy wording, proxy ratings may differ from target-person self-reports, particularly for perceived knowledge and self-reported practice. The proxy-excluded sensitivity analysis showed estimates in the same direction for internal control experience but attenuated statistical evidence, indicating that proxy response status should be considered when interpreting the findings.

## Conclusion

Among adults aged 80 years and older with valid self-reported or proxy-reported knowledge and practice data, the two measures were largely concordant. A positive knowledge–practice discrepancy was observed in a minority and co-occurred with a less favorable functional and psychosocial profile. Internal control experience was the most consistent cross-sectional correlate of discrepancy presence and magnitude in the full analytical sample, but this association was attenuated after excluding proxy interviews. These findings identify perceived control as a potential marker of broader knowledge–practice alignment, but longitudinal and behavior-specific studies are needed before causal or intervention-related conclusions can be drawn.

## Data Availability

Publicly available datasets were analyzed in this study. This data can be found at: www.dza.de/forschung/fdz/d80.

## References

[1] Marshall N Butler M Lambert V Timon CM Joyce D Warters A. Health literacy interventions and health literacy-related outcomes for older adults: a systematic review. BMC Health Serv Res. (2025) 25:319. doi: 10.1186/s12913-025-12457-7, 40011860 PMC11863724

[2] Schönfeld MS Pfisterer-Heise S Bergelt C. Self-reported health literacy and medication adherence in older adults: a systematic review. BMJ Open. (2021) 11:e056307. doi: 10.1136/bmjopen-2021-056307, 34916329 PMC8679075

[3] Xie L Zhang S Xin M Zhu M Lu W Mo PK. Electronic health literacy and health-related outcomes among older adults: a systematic review. Prev Med. (2022) 157:106997. doi: 10.1016/j.ypmed.2022.106997, 35189203

[4] Cochrane LJ Olson CA Murray S Dupuis M Tooman T Hayes S. Gaps between knowing and doing: understanding and assessing the barriers to optimal health care. J Contin Educ Heal Prof. (2007) 27:94–102. doi: 10.1002/chp.106, 17576625

[5] Graham ID Logan J Harrison MB Straus SE Tetroe J Caswell W . Lost in knowledge translation: time for a map? J Contin Educ Heal Prof. (2006) 26:13–24. doi: 10.1002/chp.47, 16557505

[6] Bayati T Dehghan A Bonyadi F Bazrafkan L. Investigating the effect of education on health literacy and its relation to health-promoting behaviors in health center. J Educ Health Promot. (2018) 7:127. doi: 10.4103/jehp.jehp_65_18, 30505855 PMC6225398

[7] Schönenberg A Mühlhammer HM Lehmann T Prell T. Adherence to medication in Neurogeriatric patients: insights from the NeuroGerAd study. J Clin Med. (2022) 11:5353. doi: 10.3390/jcm11185353, 36143000 PMC9501565

[8] Brown MT Bussell J Dutta S Davis K Strong S Mathew S. Medication adherence: truth and consequences. Am J Med Sci. (2016) 351:387–99. doi: 10.1016/j.amjms.2016.01.010, 27079345

[9] Brown MT Bussell JK. Medication adherence: WHO cares? Mayo Clin Proc. (2011) 86:304–14. doi: 10.4065/mcp.2010.0575, 21389250 PMC3068890

[10] Chen Y Gao J Lu M. Medication adherence trajectory of patients with chronic diseases and its influencing factors: a systematic review. J Adv Nurs. (2024) 80:11–41. doi: 10.1111/jan.15776, 37408103

[11] Ruksakulpiwat S Schiltz NK Irani E Josephson RA Adams J Still CH. Medication adherence of older adults with hypertension: a systematic review. Patient Prefer Adherence. (2024) 18:957–75. doi: 10.2147/PPA.S459678, 38737487 PMC11088410

[12] Simpson SH Eurich DT Majumdar SR Padwal RS Tsuyuki RT Varney J . A meta-analysis of the association between adherence to drug therapy and mortality. BMJ. (2006) 333:15. doi: 10.1136/bmj.38875.675486.55, 16790458 PMC1488752

[13] Mendorf S Witte OW Grosskreutz J Zipprich HM Prell T. What predicts different kinds of nonadherent behavior in elderly people with Parkinson's disease? Front Med (Lausanne). (2020) 7:103. doi: 10.3389/fmed.2020.00103, 32269998 PMC7109286

[14] Stewart S-JF Moon Z Horne R. Medication nonadherence: health impact, prevalence, correlates and interventions. Psychol Health. (2023) 38:726–65. doi: 10.1080/08870446.2022.2144923, 36448201

[15] Baby B McKinnon A Patterson K Patel H Sharma R Carter C . Tools to measure barriers to medication management capacity in older adults: a scoping review. BMC Geriatr. (2024) 24:285. doi: 10.1186/s12877-024-04893-7, 38532328 PMC10967066

[16] Hepburn J Williams L McCann L. Barriers to and facilitators of digital health technology adoption among older adults with chronic diseases: updated systematic review. JMIR Aging. (2025) 8:e80000. doi: 10.2196/80000, 40934502 PMC12464506

[17] Michaelsen MM Esch T. Functional mechanisms of health behavior change techniques: a conceptual review. Front Psychol. (2022) 13:725644. doi: 10.3389/fpsyg.2022.725644, 35369223 PMC8973264

[18] Michaelsen MM Esch T. Understanding health behavior change by motivation and reward mechanisms: a review of the literature. Front Behav Neurosci. (2023) 17:1151918. doi: 10.3389/fnbeh.2023.1151918, 37405131 PMC10317209

[19] Wang LY Hu ZY Chen HX Zhu H Zhou CF Zhang RX . Systematic review of longitudinal studies on daily health behavior and activity of daily living among older adults. Front Public Health. (2025) 13:1419279. doi: 10.3389/fpubh.2025.1419279, 40046125 PMC11879806

[20] Schönenberg A Heimrich KG Prell T. Impact of depressive symptoms on medication adherence in older adults with chronic neurological diseases. BMC Psychiatry. (2024) 24:131. doi: 10.1186/s12888-024-05585-7, 38365646 PMC10870557

[21] Ahmed M Cerda I Maloof M. Breaking the vicious cycle: the interplay between loneliness, metabolic illness, and mental health. Front Psychol. (2023) 14:1134865. doi: 10.3389/fpsyt.2023.1134865, 36970267 PMC10030736

[22] Wurm S Diehl M Kornadt AE Westerhof GJ Wahl HW. How do views on aging affect health outcomes in adulthood and late life? Explanations for an established connection. Dev Rev. (2017) 46:27–43. doi: 10.1016/j.dr.2017.08.002, 33927468 PMC8081396

[23] Lachman ME Neupert SD Agrigoroaei S. "Chapter 11 - the relevance of control beliefs for health and aging". In: Schaie KW Willis SL, editors. Handbook of the Psychology of Aging, Seventh Edn. San Diego: Academic Press (2011). p. 175–90.

[24] Albrecht A Kaspar R Simonson J Stuth S Hameister N. Old Age in Germany (D80+): Representative Survey 2020. Scientific Use File Version 1.0. Berlin: Research Data Centre of the German Centre of Gerontology (2022).

[25] Stirratt MJ Dunbar-Jacob J Crane HM Simoni JM Czajkowski S Hilliard ME . Self-report measures of medication adherence behavior: recommendations on optimal use. Transl Behav Med. (2015) 5:470–82. doi: 10.1007/s13142-015-0315-2, 26622919 PMC4656225

[26] Michie S van Stralen MM West R. The behaviour change wheel: a new method for characterising and designing behaviour change interventions. Implement Sci. (2011) 6:42. doi: 10.1186/1748-5908-6-42, 21513547 PMC3096582

[27] Dineen-Griffin S Garcia-Cardenas V Williams K Benrimoj SI. Helping patients help themselves: a systematic review of self-management support strategies in primary health care practice. PLoS One. (2019) 14:e0220116. doi: 10.1371/journal.pone.0220116, 31369582 PMC6675068

[28] Menec VH Chipperfield JG. The interactive effect of perceived control and functional status on health and mortality among young-old and old-old adults. J Gerontol B Psychol Sci Soc Sci. (1997) 52:P118–26. 9158563 10.1093/geronb/52b.3.p118

[29] Lachman ME Agrigoroaei S. Promoting functional health in midlife and old age: long-term protective effects of control beliefs, social support, and physical exercise. PLoS One. (2010) 5:e13297. doi: 10.1371/journal.pone.0013297, 20949016 PMC2952603

[30] Robinson SA Lachman ME. Perceived control and aging: a Mini-review and directions for future research. Gerontology. (2017) 63:435–42. doi: 10.1159/000468540, 28391279 PMC5561527

[31] Hagger MS Luszczynska A. Implementation intention and action planning interventions in health contexts: state of the research and proposals for the way forward. Appl Psychol Health Well Being. (2014) 6:1–47. doi: 10.1111/aphw.12017, 24591064

[32] Zhang CQ Zhang R Schwarzer R Hagger MS. A meta-analysis of the health action process approach. Health Psychol. (2019) 38:623–37. doi: 10.1037/hea0000728, 30973747

[33] Vermunt N Harmsen M Westert GP Olde Rikkert MGM Faber MJ. Collaborative goal setting with elderly patients with chronic disease or multimorbidity: a systematic review. BMC Geriatr. (2017) 17:167. doi: 10.1186/s12877-017-0534-0, 28760149 PMC5537926

[34] Ahmed S Heaven A Lawton R Rawlings G Sloan C Clegg A. Behaviour change techniques in personalised care planning for older people: a systematic review. Br J Gen Pract. (2021) 71:e121–7. doi: 10.3399/bjgp20X714017, 33495201 PMC7846352

[35] Palacio A Garay D Langer B Taylor J Wood BA Tamariz L. Motivational interviewing improves medication adherence: a systematic review and Meta-analysis. J Gen Intern Med. (2016) 31:929–40. doi: 10.1007/s11606-016-3685-3, 27160414 PMC4945560

