# Supplementary material for: Knowledge–practice concordance and discrepancies in very old adults
Source: Front Med (Lausanne). 2026 Aug 24;13:1891660. doi: 10.3389/fmed.2026.1891660 (PMC13547929; doi:10.3389/fmed.2026.1891660)
Supplement: Supplementary file 1 [file Data_Sheet_1.DOCX]

# Supplement

## Supplementary Table S1: Participant flow and model-specific analytical sample sizes

| Section | Item | N | Excluded n | Positive-discrepancy events |
| --- | --- | --- | --- | --- |
| Participant flow | Original D80+ Scientific Use File | 10,578 | — | — |
|  | Missing health-related knowledge item | 7,509 | — | — |
|  | Valid health-related knowledge data | 3,069 | — | — |
|  | Knowledge = “never”; practice not administered | 118 | — | — |
|  | Other missing practice data among eligible respondents | 32 | — | — |
|  | Valid health-related knowledge and practice data | 2,919 | — | — |
|  | Missing or non-positive calibrated weight | 0 | — | — |
|  | Missing PSU identifier | 0 | — | — |
|  | Missing stratum identifier | 0 | — | — |
|  | Final sample eligible for CATI2-calibrated complex-survey analyses | 2,919 | — | — |
| Model-specific analytical samples | Model 1: sociodemographic | 2,847 | 72 | 335 |
|  | Model 2: + clinical-functional | 2,708 | 211 | 321 |
|  | Model 3: + psychosocial | 2,324 | 595 | 280 |

**Note.** The practice item was administered only to participants who reported at least rare health-related knowledge. Therefore, participants selecting the lowest knowledge category (“never”) had structurally missing practice data and were not eligible for the knowledge–practice analyses. All participants in the analytical sample had valid positive CATI2 calibrated weights, primary sampling unit identifiers, and stratum information. Model-specific samples were based on complete cases for the variables included in each hierarchical model. Model 1 included age, sex, and education. Model 2 additionally included pain, multimorbidity, basic activities of daily living, and instrumental activities of daily living. Model 3 additionally included loneliness, depressive symptoms, life satisfaction, positive and negative aging experiences, and internal and external control experience. Positive-discrepancy events denote participants whose reported health-related knowledge exceeded their self-reported health-related practice. PSU = primary sampling unit.

## Supplementary Table S2. Variables used in the analyses

| **Variable in manuscript** | **Original D80+ variable** | **Operationalization / coding** |
| --- | --- | --- |
| **Core variables and gap definitions** | | |
| Health-related knowledge | geskom1_c2 | The item was administered in D80+ CATI Module 2. The original German wording for interviews with the target person was: “Wie häufig wissen Sie, was Sie machen müssen, um gesund zu bleiben, wieder gesund zu werden oder Ihre Gesundheit zu stärken?” For proxy interviews, the wording was: “Wie häufig weiß Herr/Frau [Name ZP], was er/sie machen muss, um gesund zu bleiben, wieder gesund zu werden oder seine/ihre Gesundheit zu stärken?” Interviewers were instructed to read the response options aloud and to enter a spontaneous answer directly if appropriate. The English wording used in the manuscript follows the official D80+ English instrument documentation: “How often do you know what you need to do to stay healthy, get healthy again or strengthen your health?”  **Response options and coding:** 1 = never (“Nie”); 2 = rather rarely (“Eher selten”); 3 = sometimes (“Manchmal”); 4 = frequently (“Häufig”). Higher values indicate greater perceived general health-related knowledge.  **Availability, skip logic, and missingness:** The item was part of CATI Module 2, which was available for 3,233 of the 10,578 participants in the Scientific Use File. Valid responses were available for 3,069 participants. In the full Scientific Use File, 7,509 values were unavailable: 7,345 because CATI Module 2 was not administered, 152 because the respondent answered “don’t know”, and 12 because the respondent refused. Thus, 164 of the 3,233 CATI Module 2 participants had no valid knowledge response. |
| Self-reported health-related practice | geskom2_c2 | **Original wording and context:** This item immediately followed the knowledge item in D80+ CATI Module 2. The wording referred back to what respondents had just reported knowing about maintaining, restoring, or strengthening their health. The original German wording for interviews with the target person was: “Wie häufig halten Sie sich auch daran?” For proxy interviews, the wording was: “Wie häufig hält er/sie sich auch daran?” Interviewers were instructed to read the response options aloud and to enter a spontaneous answer directly if appropriate. The official D80+ English instrument documentation translates the target-person item as: “How often do you comply?” Because the brief wording refers back to the preceding knowledge item, it was interpreted in this study as broad self-reported practice consistent with perceived health-related knowledge; it did not refer to a specific recommendation, behavior, disease, or treatment.  **Response options and coding:** 1 = never (“Nie”); 2 = rather rarely (“Eher selten”); 3 = sometimes (“Manchmal”); 4 = frequently (“Häufig”). Higher values indicate more frequent self-reported practice.  **Availability, skip logic, and missingness:** The item was administered only if the knowledge response was 2, 3, or 4. It was therefore structurally not applicable to the 118 participants who selected “never” for knowledge. Among the 2,951 participants eligible for the practice item, 2,919 had a valid response and 32 had another missing practice value. In the full Scientific Use File, 7,659 practice values were unavailable: 7,345 because CATI Module 2 was not administered, 118 because of the programmed skip after a knowledge response of “never”, 164 because no valid knowledge response was available, and 32 because of additional missing practice data among eligible participants. |
| Positive knowledge–practice discrepancy, presence | Derived variable | Binary indicator coded 1 if Knowledge > Practice and 0 otherwise. |
| Positive knowledge–practice discrepancy, magnitude | Derived variable | Calculated as max(Knowledge − Practice, 0). Participants without a gap were assigned 0. |
| **Sociodemographic variables** | | |
| Age | alter_int | Age in years. |
| Sex | gender | Original dataset categories; included as categorical variable. |
| Education | isced | International Standard Classification of Education, three categories: lower secondary or below, upper secondary, tertiary education. |
| **Clinical and functional variables** | | |
| Pain | schmerz | Original ordinal response categories; higher values indicate greater pain burden. |
| Multimorbidity | multmor_m22 | Mean score across 22 doctor-treated conditions; range 0–1. Higher values indicate greater multimorbidity. |
| Basic ADL | funkges_m1_c2 | Mean score across seven basic activities of daily living; range 0–2. Higher values indicate greater independence. |
| Instrumental ADL | funkges_m2 | Mean score across seven instrumental activities of daily living; range 0–2. Higher values indicate greater independence. |
| **Psychosocial variables** | | |
| Loneliness | einsam | Frequency of loneliness during the past week: 1 = never or almost never, 2 = sometimes, 3 = mostly, 4 = always or almost always. Higher values indicate more frequent loneliness. |
| Depressive symptoms | dias4 | DIA-S4 sum score based on four dichotomous depressive symptom items; range 0–4. Higher values indicate greater depressive symptom burden. |
| Life satisfaction | zufri | Continuous measure of general life satisfaction; higher values indicate greater satisfaction. |
| Positive aging experience | alterl_m1 | Mean score of five items rated from 1 to 5. Higher values indicate stronger positive awareness of age-related changes. |
| Negative aging experience | alterl_m2 | Mean score of five items rated from 1 to 5. Higher values indicate stronger negative awareness of age-related changes. |
| Internal control experience | eikontr_m1_c2 | Mean score of two items assessing internal control experience ranging from 1 to 4; higher values indicate stronger internal control beliefs. |
| External control experience | eikontr_m2_c2 | Mean score of two items assessing external control experience ranging from 1 to 4; higher values indicate stronger external control beliefs. |

**Note.** ADL = activities of daily living; IADL = instrumental activities of daily living; DIA-S4 = short depression scale used in the D80+ study. The primary outcome was the presence of a positive knowledge–practice discrepancy, defined as reported health-related knowledge exceeding reported health-related practice. The German wording shown above is the wording administered in the D80+ study. The English wording follows the publicly available D80+ English research-instrument documentation. That documentation states that it was automatically translated from German into English using DeepL without additional checking or revision; the English version should therefore be regarded as a documentary translation rather than a formally validated translation. Analyses were based on the original German-language study data and coding. Missingness counts refer to the unweighted D80+ Scientific Use File. CATI = computer-assisted telephone interview; ZP = target person; PX = proxy respondent.

## Supplementary Table S3: Observed-only comparison of participants included in and excluded from the fully adjusted model

| Variable | Category | Included in Model 3 | Excluded from Model 3 | SMD |
| --- | --- | --- | --- | --- |
| Age, years |  | 85.30 (4.12) | 85.66 (4.08) | 0.09 |
| Sex | Male | 40.0% | 30.7% | 0.13 |
|  | Female | 60.0% | 69.3% |  |
|  | Diverse | 0.0% | 0.0% |  |
| Education | Lower secondary or below | 21.0% | 28.8% | 0.10 |
|  | Upper secondary | 50.6% | 48.0% |  |
|  | Tertiary | 28.4% | 23.2% |  |
| Pain | No pain | 21.4% | 22.5% | 0.03 |
|  | Mild | 26.9% | 26.3% |  |
|  | Moderate | 32.3% | 31.0% |  |
|  | Severe | 16.5% | 18.4% |  |
|  | Very severe | 3.0% | 1.8% |  |
| Multimorbidity |  | 0.23 (0.14) | 0.22 (0.14) | 0.10 |
| Basic ADL |  | 1.72 (0.45) | 1.71 (0.46) | <0.01 |
| Instrumental ADL |  | 1.51 (0.60) | 1.50 (0.62) | 0.02 |
| Loneliness | Never or almost never | 51.9% | 45.4% | 0.08 |
|  | Sometimes | 39.9% | 44.4% |  |
|  | Mostly | 6.4% | 8.4% |  |
|  | Always or almost always | 1.8% | 1.7% |  |
| Depressive symptoms |  | 1.26 (1.26) | 1.36 (1.31) | 0.08 |
| Life satisfaction |  | 7.34 (1.98) | 7.39 (2.04) | 0.03 |
| Positive aging experience |  | 3.33 (0.69) | 3.23 (0.81) | 0.14 |
| Negative aging experience |  | 2.83 (0.87) | 2.78 (0.90) | 0.07 |
| Internal control experience |  | 3.25 (0.67) | 3.17 (0.74) | 0.10 |
| External control experience |  | 1.95 (0.69) | 1.98 (0.70) | 0.05 |
| Positive knowledge–practice discrepancy | No positive discrepancy | 87.6% | 88.5% | 0.01 |
|  | Positive discrepancy | 12.4% | 11.5% |  |

**Note.** Values are CATI2-calibrated complex-survey-weighted means (standard deviations) or weighted percentages. Unweighted group sizes were 2,324 participants included in the fully adjusted Model 3 and 595 participants excluded because of missing covariate data. Comparisons were observed-only: for each variable, comparisons were restricted to participants with an observed value for that variable, and missing values were not treated as a separate category. Accordingly, the number of valid excluded participants varied across variables: education, n=523; pain, n=527; multimorbidity, n=540; basic ADL, n=594; instrumental ADL, n=566; loneliness, n=525; depressive symptoms, n=308; life satisfaction, n=526; positive aging experience, n=568; negative aging experience, n=561; internal control experience, n=580; and external control experience, n=577. All included participants had complete data for the variables in Model 3. Standardized mean differences (SMDs) describe the magnitude of observed group differences. Values of approximately 0.10, 0.20, and 0.50 may be interpreted as small, moderate, and large differences, respectively. ADL = activities of daily living; IADL = instrumental activities of daily living.

## Supplementary Table S4 Missing data and model-specific complete-case sample sizes

| Section | Variable or model | Total N | Valid or complete n | Missing or incomplete n | Missing or incomplete % |
| --- | --- | --- | --- | --- | --- |
| Variable-specific missingness | Age | 2,919 | 2,919 | 0 | 0.0 |
|  | Sex | 2,919 | 2,919 | 0 | 0.0 |
|  | Education | 2,919 | 2,847 | 72 | 2.5 |
|  | Pain | 2,919 | 2,851 | 68 | 2.3 |
|  | Multimorbidity | 2,919 | 2,864 | 55 | 1.9 |
|  | Basic ADL | 2,919 | 2,918 | 1 | 0.0 |
|  | Instrumental ADL | 2,919 | 2,890 | 29 | 1.0 |
|  | Loneliness | 2,919 | 2,849 | 70 | 2.4 |
|  | Depressive symptoms | 2,919 | 2,632 | 287 | 9.8 |
|  | Life satisfaction | 2,919 | 2,850 | 69 | 2.4 |
|  | Positive aging experience | 2,919 | 2,892 | 27 | 0.9 |
|  | Negative aging experience | 2,919 | 2,885 | 34 | 1.2 |
|  | Internal control experience | 2,919 | 2,904 | 15 | 0.5 |
|  | External control experience | 2,919 | 2,901 | 18 | 0.6 |
| Model-specific complete cases | Model 1: sociodemographic | 2,919 | 2,847 | 72 | 2.5 |
|  | Model 2: + clinical-functional | 2,919 | 2,708 | 211 | 7.2 |
|  | Model 3: + psychosocial | 2,919 | 2,324 | 595 | 20.4 |

**Note**. Variable-specific missingness was assessed within the analytical sample of 2,919 participants with valid health-related knowledge and self-reported practice data. Model-specific samples were based on complete cases for all variables included in the respective hierarchical model. Model 1 included age, sex, and education. Model 2 additionally included pain, multimorbidity, basic activities of daily living, and instrumental activities of daily living. Model 3 additionally included loneliness, depressive symptoms, life satisfaction, positive and negative aging experiences, and internal and external control experience. Health-related knowledge, self-reported practice, the derived discrepancy outcomes, the CATI2 calibrated analysis weight, primary sampling unit, and survey stratum were complete in all 2,919 participants. Percentages may not correspond exactly to the displayed counts because of rounding. ADL = activities of daily living; IADL = instrumental activities of daily living.

## Supplementary Table S5. Hierarchical complex survey logistic regression models for positive knowledge–practice discrepancy

| Predictor | Model 1, OR (95% CI), p | Model 2, OR (95% CI), p | Model 3, OR (95% CI), p |
| --- | --- | --- | --- |
| Age | 1.04 (1.00–1.08), .075 | 1.00 (0.96–1.05), .842 | 1.01 (0.97–1.06), .547 |
| Female | 1.04 (0.76–1.41), .824 | 0.95 (0.68–1.33), .784 | 0.92 (0.63–1.34), .670 |
| Education: upper secondary | 0.80 (0.51–1.27), .344 | 0.86 (0.56–1.33), .494 | 0.93 (0.58–1.48), .747 |
| Education: tertiary | 0.78 (0.48–1.28), .327 | 0.92 (0.57–1.48), .720 | 0.99 (0.59–1.65), .955 |
| Pain: mild | — | 1.04 (0.64–1.71), .865 | 0.86 (0.51–1.45), .567 |
| Pain: moderate | — | 1.24 (0.76–2.01), .391 | 0.91 (0.54–1.53), .709 |
| Pain: severe | — | 1.62 (0.90–2.93), .111 | 1.21 (0.64–2.28), .552 |
| Pain: very severe | — | 1.02 (0.34–3.11), .966 | 0.83 (0.27–2.54), .747 |
| Multimorbidity | — | 0.65 (0.17–2.46), .530 | 0.55 (0.14–2.14), .390 |
| Basic ADL | — | 0.60 (0.35–1.02), .058 | 0.87 (0.47–1.62), .662 |
| Instrumental ADL | — | 0.87 (0.56–1.35), .532 | 1.19 (0.69–2.04), .532 |
| Loneliness: sometimes | — | — | 1.09 (0.77–1.54), .645 |
| Loneliness: mostly | — | — | 1.08 (0.54–2.16), .820 |
| Loneliness: always or almost always | — | — | 0.61 (0.16–2.33), .470 |
| Depressive symptoms | — | — | 1.12 (0.94–1.34), .192 |
| Life satisfaction | — | — | 0.94 (0.85–1.05), .269 |
| Positive aging experience | — | — | 1.00 (0.77–1.29), .988 |
| Negative aging experience | — | — | 1.23 (0.96–1.58), .096 |
| Internal control experience | — | — | 0.69 (0.53–0.89), .005 |
| External control experience | — | — | 0.99 (0.76–1.30), .964 |
| Global Wald test | F(4,1641)=1.15, p=.330 | F(11,1584)=1.37, p=.179 | F(20,1426)=1.87, p=.011 |

**Note.** Estimates are from complex-survey logistic regression models predicting the presence of a positive knowledge–practice discrepancy, defined as reported health-related knowledge exceeding self-reported health-related practice. Models used the CATI2 calibrated weighting factor because the knowledge and practice items were administered in CATI Module 2. Models additionally accounted for primary sampling units and survey strata; variances were estimated using Taylor-series linearization. Model 1 included age, sex, and education (N=2,847; 335 positive-discrepancy events). Model 2 additionally included pain, multimorbidity, basic ADL, and instrumental ADL (N=2,708; 321 events). Model 3 additionally included loneliness, depressive symptoms, life satisfaction, positive and negative aging experiences, and internal and external control experience (N=2,324; 280 events). Reference categories were male sex, lower secondary education or below, no pain, and loneliness “never or almost never.” Odds ratios below 1 indicate lower odds of a positive knowledge–practice discrepancy. ADL = activities of daily living; IADL = instrumental activities of daily living.

## Supplementary Table S6. Hierarchical complex-survey linear regression models for positive knowledge–practice discrepancy magnitude

| Predictor | Model 1, B (95% CI), p | Model 2, B (95% CI), p | Model 3, B (95% CI), p |
| --- | --- | --- | --- |
| Age | 0.005 (−0.000 to 0.011), .065 | 0.001 (−0.004 to 0.007), .623 | 0.002 (−0.004 to 0.008), .481 |
| Female | 0.002 (−0.035 to 0.039), .901 | −0.009 (−0.048 to 0.030), .655 | −0.015 (−0.060 to 0.030), .512 |
| Education: upper secondary | −0.027 (−0.085 to 0.032), .370 | −0.020 (−0.076 to 0.037), .493 | −0.007 (−0.068 to 0.053), .815 |
| Education: tertiary | −0.019 (−0.083 to 0.046), .568 | −0.002 (−0.066 to 0.062), .951 | 0.011 (−0.058 to 0.079), .763 |
| Pain: mild | — | 0.008 (−0.048 to 0.063), .790 | −0.016 (−0.077 to 0.044), .599 |
| Pain: moderate | — | 0.029 (−0.024 to 0.081), .287 | −0.012 (−0.072 to 0.048), .691 |
| Pain: severe | — | 0.065 (−0.011 to 0.141), .092 | 0.031 (−0.049 to 0.110), .453 |
| Pain: very severe | — | −0.013 (−0.133 to 0.107), .832 | −0.040 (−0.169 to 0.088), .540 |
| Multimorbidity | — | −0.060 (−0.216 to 0.095), .446 | −0.083 (−0.242 to 0.076), .307 |
| Basic ADL | — | −0.074 (−0.164 to 0.015), .104 | −0.029 (−0.122 to 0.065), .546 |
| Instrumental ADL | — | −0.018 (−0.081 to 0.046), .583 | 0.016 (−0.056 to 0.088), .664 |
| Loneliness: sometimes | — | — | 0.017 (−0.026 to 0.060), .434 |
| Loneliness: mostly | — | — | 0.015 (−0.088 to 0.118), .772 |
| Loneliness: always or almost always | — | — | −0.086 (−0.235 to 0.063), .259 |
| Depressive symptoms | — | — | 0.018 (−0.007 to 0.043), .151 |
| Life satisfaction | — | — | −0.011 (−0.028 to 0.007), .225 |
| Positive aging experience | — | — | 0.005 (−0.026 to 0.036), .734 |
| Negative aging experience | — | — | 0.015 (−0.015 to 0.044), .326 |
| Internal control experience | — | — | −0.055 (−0.098 to −0.012), .012 |
| External control experience | — | — | −0.004 (−0.041 to 0.033), .820 |
| Global Wald test | F(4,1641)=1.07, p=.369 | F(11,1584)=1.08, p=.370 | F(20,1426)=1.44, p=.096 |

**Note.** Estimates are from complex-survey linear regression models predicting the magnitude of the positive knowledge–practice discrepancy, calculated as max(knowledge − practice, 0). The score ranged from 0 to 3, with higher values indicating a larger positive discrepancy. Because the outcome was derived from two ordinal items, was bounded, and contained a high proportion of zero values, these models were interpreted as secondary, pragmatic analyses. Models used the CATI2 calibrated weighting factor because the knowledge and practice items were administered in CATI Module 2. Models additionally accounted for primary sampling units and survey strata; variances were estimated using Taylor-series linearization. Model 1 included age, sex, and education (N=2,847). Model 2 additionally included pain, multimorbidity, basic ADL, and instrumental ADL (N=2,708). Model 3 additionally included loneliness, depressive symptoms, life satisfaction, positive and negative aging experiences, and internal and external control experience (N=2,324). Reference categories were male sex, lower secondary education or below, no pain, and loneliness “never or almost never.” Negative coefficients indicate a smaller positive knowledge–practice discrepancy magnitude. ADL = activities of daily living; IADL = instrumental activities of daily living.

## Supplementary Table S7. Ordinal sensitivity analysis of positive knowledge–practice discrepancy magnitude

| Predictor | OR | 95% CI | p |
| --- | --- | --- | --- |
| Age | 1.02 | 0.97–1.06 | .508 |
| Female | 0.92 | 0.63–1.32 | .639 |
| Education: upper secondary | 0.92 | 0.58–1.47 | .740 |
| Education: tertiary | 1.02 | 0.61–1.71 | .949 |
| Pain: mild | 0.88 | 0.52–1.51 | .650 |
| Pain: moderate | 0.91 | 0.54–1.52 | .706 |
| Pain: severe | 1.24 | 0.67–2.29 | .498 |
| Pain: very severe | 0.81 | 0.27–2.36 | .694 |
| Multimorbidity | 0.53 | 0.14–2.02 | .355 |
| Basic ADL | 0.86 | 0.46–1.59 | .621 |
| Instrumental ADL | 1.18 | 0.69–2.02 | .541 |
| Loneliness: sometimes | 1.11 | 0.78–1.57 | .562 |
| Loneliness: mostly | 1.08 | 0.55–2.11 | .833 |
| Loneliness: always or almost always | 0.60 | 0.16–2.19 | .437 |
| Depressive symptoms | 1.12 | 0.94–1.34 | .200 |
| Life satisfaction | 0.93 | 0.83–1.05 | .242 |
| Positive aging experience | 1.02 | 0.78–1.33 | .881 |
| Negative aging experience | 1.22 | 0.95–1.55 | .115 |
| Internal control experience | 0.68 | 0.52–0.89 | .005 |
| External control experience | 0.98 | 0.73–1.30 | .877 |

**Note.** Estimates are from a fully adjusted complex-survey proportional-odds model accounting for the calibrated D80+ analysis weight (CATI2 calibrated weighting factor), primary sampling units, and survey strata. The ordinal outcome represented the magnitude of the positive knowledge–practice discrepancy and comprised three categories: no positive discrepancy, a one-category discrepancy, and a discrepancy of at least two response categories. The model included 2,324 participants: 2,044 with no positive discrepancy, 239 with a one-category discrepancy, and 41 with a discrepancy of at least two categories. Odds ratios above 1 indicate higher odds of belonging to a higher discrepancy category; odds ratios below 1 indicate lower odds. The model was adjusted for age, sex, education, pain, multimorbidity, basic and instrumental activities of daily living, loneliness, depressive symptoms, life satisfaction, positive and negative aging experiences, and internal and external control experience. Reference categories were male sex, lower secondary education or below, no pain, and loneliness “never or almost never.” ADL = activities of daily living; IADL = instrumental activities of daily living.

## Supplementary Table S8: Design-based directional contrasts of knowledge–practice patterns

**Panel A. Knowledge > practice versus exact concordance**

| Predictor | OR | 95% CI | p |
| --- | --- | --- | --- |
| Age | 1.02 | 0.97–1.07 | .461 |
| Female | 0.85 | 0.58–1.24 | .391 |
| Education: upper secondary | 0.92 | 0.57–1.49 | .736 |
| Education: tertiary | 0.91 | 0.54–1.54 | .733 |
| Pain: mild | 0.86 | 0.50–1.46 | .571 |
| Pain: moderate | 0.94 | 0.55–1.61 | .814 |
| Pain: severe | 1.18 | 0.61–2.30 | .616 |
| Pain: very severe | 0.78 | 0.25–2.40 | .661 |
| Multimorbidity | 0.43 | 0.11–1.71 | .234 |
| Basic ADL | 0.99 | 0.51–1.91 | .979 |
| Instrumental ADL | 1.12 | 0.64–1.96 | .683 |
| Loneliness: sometimes | 1.13 | 0.79–1.62 | .498 |
| Loneliness: mostly | 1.24 | 0.63–2.46 | .532 |
| Loneliness: always or almost always | 0.58 | 0.15–2.21 | .422 |
| Depressive symptoms | 1.13 | 0.94–1.34 | .188 |
| Life satisfaction | 0.95 | 0.85–1.06 | .342 |
| Positive aging experience | 1.02 | 0.78–1.32 | .906 |
| Negative aging experience | 1.31 | 1.02–1.68 | .038 |
| Internal control experience | 0.66 | 0.51–0.85 | .001 |
| External control experience | 1.01 | 0.77–1.32 | .940 |

**Panel B. Practice > knowledge versus exact concordance**

| Predictor | OR | 95% CI | p |
| --- | --- | --- | --- |
| Age | 1.04 | 0.98–1.09 | .171 |
| Female | 0.48 | 0.32–0.70 | <.001 |
| Education: upper secondary | 0.92 | 0.54–1.57 | .765 |
| Education: tertiary | 0.71 | 0.39–1.27 | .247 |
| Pain: mild | 1.25 | 0.73–2.14 | .416 |
| Pain: moderate | 1.31 | 0.78–2.18 | .305 |
| Pain: severe | 0.97 | 0.45–2.09 | .946 |
| Pain: very severe | 0.96 | 0.28–3.35 | .955 |
| Multimorbidity | 0.19 | 0.04–0.88 | .033 |
| Basic ADL | 1.95 | 0.97–3.90 | .060 |
| Instrumental ADL | 0.68 | 0.38–1.22 | .195 |
| Loneliness: sometimes | 1.36 | 0.89–2.09 | .157 |
| Loneliness: mostly | 2.18 | 0.99–4.78 | .052 |
| Loneliness: always or almost always | 0.39 | 0.05–3.20 | .383 |
| Depressive symptoms | 1.00 | 0.83–1.20 | .977 |
| Life satisfaction | 1.01 | 0.90–1.15 | .826 |
| Positive aging experience | 1.10 | 0.85–1.42 | .486 |
| Negative aging experience | 1.39 | 1.05–1.82 | .020 |
| Internal control experience | 0.71 | 0.54–0.94 | .017 |
| External control experience | 1.08 | 0.84–1.39 | .533 |

**Note.** Separate fully adjusted complex-survey logistic regression models were fitted for each directional contrast, with exact knowledge–practice concordance as the reference category. Models accounted for the calibrated D80+ analysis weight, primary sampling units, and survey strata. Panel A compared participants with health-related knowledge exceeding self-reported practice with participants showing exact concordance and included 2,054 participants, of whom 280 had a positive knowledge–practice discrepancy. Panel B compared participants with self-reported practice exceeding health-related knowledge with those showing exact concordance and included 2,044 participants, of whom 270 showed reverse discordance. Both models were adjusted for age, sex, education, pain, multimorbidity, basic and instrumental activities of daily living, loneliness, depressive symptoms, life satisfaction, positive and negative aging experiences, and internal and external control experience. Reference categories were male sex, lower secondary education or below, no pain, and loneliness “never or almost never.” Odds ratios above 1 indicate higher odds of the respective discordance pattern relative to exact concordance. ADL = activities of daily living; IADL = instrumental activities of daily living.

## Supplementary Table S9. Proxy response counts and proxy-excluded sensitivity analysis

**Panel A. Proxy response counts**

| **Sample** | **Target person, n (%)** | **Proxy interview, n (%)** |
| --- | --- | --- |
| Analytical sample with valid knowledge and practice data | 2,627 (90.0) | 292 (10.0) |
| Fully adjusted Model 3 complete-case sample | 2,099 (90.3) | 225 (9.7) |

**Panel B. Proxy-excluded sensitivity analysis of the fully adjusted models**

| **Outcome** | **Model** | **N** | **Events, n** | **Internal control estimate (95% CI), p** |
| --- | --- | --- | --- | --- |
| Positive discrepancy presence | Logistic Model 3 excluding proxy interviews | 2,099 | 233 | OR 0.82 (0.61–1.09), .174 |
| Positive discrepancy magnitude | Linear Model 3 excluding proxy interviews | 2,099 | — | B −0.024 (−0.060 to 0.012), .200 |

**Note.** Proxy status was defined using the D80+ CATI Module 2 respondent variable, distinguishing target-person interviews from proxy interviews. The main analyses retained proxy responses because the D80+ instrument included corresponding proxy wording for the knowledge and practice items. Panel A reports unweighted counts and percentages. Panel B reports a sensitivity analysis excluding proxy interviews. The fully adjusted proxy-excluded models used the same covariate set as the main fully adjusted models: age, sex, education, pain, multimorbidity, basic ADL, instrumental ADL, loneliness, depressive symptoms, life satisfaction, positive and negative aging experiences, and internal and external control experience. Models used the CATI2 calibrated weighting factor and accounted for primary sampling units and survey strata. The internal-control estimate remained in the same direction as in the main analysis, but the association was attenuated and no longer statistically significant after excluding proxy interviews. ADL = activities of daily living; IADL = instrumental activities of daily living.
